# Influence of nanoparticle encapsulation and encoding on the surface chemistry of polymer carrier beads

**DOI:** 10.1038/s41598-023-38518-7

**Published:** 2023-07-24

**Authors:** Lena Scholtz, Isabella Tavernaro, J. Gerrit Eckert, Marc Lutowski, Daniel Geißler, Andreas Hertwig, Gundula Hidde, Nadja C. Bigall, Ute Resch-Genger

**Affiliations:** 1 Division 1.2 Biophotonics, Federal Institute for Materials Research and Testing (BAM), Richard-Willstätter-Str. 11, 12489 Berlin, Germany; 2 Institute for Chemistry and Biochemistry, Free University Berlin, Takustraße 3, 14195 Berlin, Germany; 3 Institute of Physical Chemistry and Electrochemistry, Leibniz University Hannover, Callinstraße 3A, 30167 Hannover, Germany; 4 Division 6.1 Surface Analysis and Interfacial Chemistry, Federal Institute for Materials Research and Testing (BAM), Unter den Eichen 87, 12205 Berlin, Germany; 5 Cluster of Excellence PhoenixD (Photonics, Optics, and Engineering − Innovation Across Disciplines), 30167 Hannover, Germany; 6 Present Address: PolyAn GmbH, Schkopauer Ring 6, 12681 Berlin, Germany

**Keywords:** Nanoscience and technology, Optics and photonics

## Abstract

Surface-functionalized polymer beads encoded with molecular luminophores and nanocrystalline emitters such as semiconductor nanocrystals, often referred to as quantum dots (QDs), or magnetic nanoparticles are broadly used in the life sciences as reporters and carrier beads. Many of these applications require a profound knowledge of the chemical nature and total number of their surface functional groups (FGs), that control bead charge, colloidal stability, hydrophobicity, and the interaction with the environment and biological systems. For bioanalytical applications, also the number of groups accessible for the subsequent functionalization with, e.g., biomolecules or targeting ligands is relevant. In this study, we explore the influence of QD encoding on the amount of carboxylic acid (COOH) surface FGs of 2 µm polystyrene microparticles (PSMPs). This is done for frequently employed oleic acid and oleylamine stabilized, luminescent core/shell CdSe QDs and two commonly used encoding procedures. This included QD addition during bead formation by a thermally induced polymerization reaction and a post synthetic swelling procedure. The accessible number of COOH groups on the surface of QD-encoded and pristine beads was quantified by two colorimetric assays, utilizing differently sized reporters and electrostatic and covalent interactions. The results were compared to the total number of FGs obtained by a conductometric titration and Fourier transform infrared spectroscopy (FTIR). In addition, a comparison of the impact of QD and dye encoding on the bead surface chemistry was performed. Our results demonstrate the influence of QD encoding and the QD-encoding strategy on the number of surface FG that is ascribed to an interaction of the QDs with the carboxylic acid groups on the bead surface. These findings are of considerable relevance for applications of nanoparticle-encoded beads and safe-by-design concepts for nanomaterials.

## Introduction

Surface-functionalized polymeric microparticles (MPs) are increasingly utilized in the life sciences. They are stained with different types of molecular and nanoscale luminophores such as organic dyes, semiconductor quantum dots (QDs), and lanthanide-based nanocrystals, or magnetic nanoparticles. Typical applications are as carrier beads for bead-based assay platforms and immune-separation, read out with fluorescence microscopy and flow cytometry^1–4^, drug carriers, conventional and multimodal labels, and fluorescent sensors^5–11^. Hydrophobic, organic dyes and nanoparticles are typically surface-capped with coordinatively bound, apolar surface ligands utilized for the initial nanomaterial synthesis. The encapsulation into organic, inorganic or hybrid particles presents an efficient and versatile strategy to render them water-dispersible and protect them from the environment^12^. This can also reduce the potential toxicity of semiconductor nanocrystals^13–16^. Particularly interesting for optical encoding and multiplexing schemes are II-VI, III-V, and IV-VI as well as doped, ternary/quaternary, and alloyed QDs^17–20^ with their unique size- and composition-dependent optical properties and high photostability^21–24^. This enables the free choice of the excitation wavelength and the simultaneous excitation of several QDs with different emission colors with a single excitation source^25–27^.

Nanoparticle (NP)-stained MPs bear functional groups (FGs) such as carboxylic acid (COOH) at their surface, which can be used for the covalent attachment of polyethylene glycol (PEG) or targeting ligands and biomolecules using, e.g., EDC/NHS chemistry, click chemistry or heterobifunctional cross-linkers^28–30^. Their fabrication requires simple procedures for bead encoding and surface functionalization. In addition, simple and cost-efficient analytical methods are needed for the characterization of their application-relevant properties and process control. Three strategies are commonly applied for the preparation of surface functionalized encoded or stained beads: (i) The addition of the staining agent before or during bead formation followed by the introduction of suitable FGs, i.e., by a grafting step, (ii) the utilization of premanufactured, surface-functionalized MPs together with a post synthetic swelling procedure, and (iii) the wrapping of beads by alternating shells of oppositely charged polyelectrolytes incorporating the staining agent (layer-by-layer (LbL) method). Procedure (i) requires NPs that survive the sometimes harsh polymerization conditions and do not undergo a loss of their functional properties such as photoluminescence^31–33^. Procedure (ii) often exploits commercial polymer beads made from, e.g., polystyrene (PS) and polymethylmethacrylate (PMMA) available in different sizes with different FGs. Challenges to be tackled particularly for the encoding with NPs like QDs include the choice of a solvent mixture for bead swelling, that does not induce the quenching of the QD photoluminescence and QD agglomeration or aggregation and prevents QD leaking. The LbL method (iii), that is not employed in this study, utilizes premanufactured polymer beads as templates coated with a NP-stained polyelectrolyte shell of varying thickness. This method is versatile, yet tedious and limited to surface staining, which can make further bioconjugation steps challenging^34–36^.

The broad application of surface-functionalized, stained MPs encouraged us to explore the influence of the particle staining procedure on the number of surface FGs by procedures (i) and (ii), exemplarily for unstained polystyrene microparticles (PSMPs) and carboxylated beads. The latter were chosen because of the frequent use of carboxylate polymer microparticles in the life sciences, e.g., for bead-based assays and DNA sequencing platforms. Aiming for a better understanding of the impact of this encoding step on the application relevant properties of such QD-stained polymer particles, we evaluated and compared both QD-staining methods in terms of the size, surface charge, amount of total and accessible FG, and luminescence properties of the resulting QD-encoded PSMPs. As representative QDs, we focused on oleic acid and oleylamine stabilized CdSe/CdS QDs with a mean particle size of 10.3 ± 1.2 nm and a strong red luminescence. Such QDs have been broadly used in many different applications. Based upon previously examined and validated protocols for FG quantification^37–40^, the total amount of (de)protonable COOH groups was determined by a conductometric titration. In addition, Fourier transform infrared (FTIR) spectroscopy was used as a semiquantitative method for the determination of the number of total COOH acid groups. The number of accessible COOH groups was obtained with an adsorption/desorption-based assay, relying on electrostatic interactions between the negatively charged beads and the relatively large, positively charged dye toluidine blue (TBO). Also, a catch-and-release assay using the small, cleavable, COOH-reactive reporter *N*-(aminoethyl)-3-(pyridin-2-yldisulfanyl)-propanamide trifluoroacetate (*N*-APPA) was performed. This involved the covalent binding of the reporter to the bead surface FGs. As control samples, we used pristine carboxylated beads as well as carboxylated beads which were encoded with rhodamine B isothiocyanate (RITC) and the neutral polarity probe Nile Red (NR) with our post synthetic swelling procedure, previously optimized for different dye classes. In addition to the quantification of the number of surface FGs, also the impact of the encoding procedure on QD luminescence was examined.

## Materials and methods

The experimental procedures employed for the synthesis of the CdSe/CdS-QDs, polyethylene glycol-*block*-poly(ε-caprolactone), the post-synthetic swelling procedures for dyes and the QD-loaded PSMPs, as well as for the fluorescence and integrating sphere spectroscopy, atomic absorption spectrometry, dynamic light scattering/zeta potential and both assays (TBO and *N*-APPA) have been partly previously employed and reported by us^31,37,38,40,41^.

### Materials

Hydrochloric acid (HCl), sodium hydroxide (NaOH), sodium dihydrogen phosphate (NaH_2_PO_4_), dimethyl sulfoxide (DMSO), acetone (≥ 99.5%), styrene (≥ 99.0%), tin(II) 2-ethylhexanoate (92.5–100%), ε-caprolactone (97%), poly(ethylene glycol) (PEG, M_W_ 2500), trioctylphosphine oxide (TOPO, 99%), 1-octadecene (ODE, 90%), toluene (≥ 99.7%), methanol (≥ 99.8%), isopropanol (≥ 99.8%), oleylamine (OLA, 70–80%), 1-octanethiol (98.5%), acrylic acid (AA, 99%, anh.), azobisisobutyronitrile (AIBN, 98%), N-(3-dimethylaminopropyl)-N'-ethylcarbodiimid hydrochloride (EDC, ≥ 98%), hydroxy-2,5-dioxopyrrolidine-3-sulfonicacid sodium salt (sulfo-NHS, 98%), Rhodamine B isothiocyanate (RITC) and Toluidine Blue O (TBO) were purchased from Sigma Aldrich. Polyvinylpyrrolidone (PVP, M_W_ 58,000), potassium bromide (KBr, p. A.), selenium powder (200 mesh, 99.999%), oleic acid (OA, 90%) and cadmium oxide (CdO, 99.998%) were obtained from Alfa Aesar. Ethanol, toluene and *n*-heptane (all spectr. grade) as well as *n*-hexane (≥ 99%) were purchased from Merck KGaA. *N*-succinimidyl-3-(2-pyridyldithio) propionate (SPDP) and tris(2-carboxyethyl) phosphine hydrochloride (TCEP) were obtained from Thermo Fisher Scientific, benzyldimethyloctadecylammonium chloride (OBDAC, 98.9%) from HPC Standards GmbH, tri-n-octylphosphine (TOP, 99.7%) and deuterated chloroform (99.8 atom%) were obtained from abcr GmbH, *n*-octadecylphosphonic acid (ODPA, > 99%) from PCI Synthesis, Nile Red (NR) from Fluka and 1-butanol (> 99.5%), tetrahydrofuran (THF, > 99.9%), N,N-dimethylformamide (DMF, > 99.9%), and chloroform from Chemsolute. All solvents used for the spectroscopy measurements and optical assays were of spectroscopic grade and all chemicals were employed without further purification. All aqueous solutions, buffers, and microparticles were prepared with deionized water (0.055 μS m^−1^; Milli-Q water, Millipore).

### Synthesis of CdSe/CdS-core/shell-QDs

The CdSe/CdS-QDs with a core/shell-architecture in hexane were prepared according to a previously described procedure^31^ adapted from Carbone et al*.*, Nightingale et al*.* and Chen et al.^21–23^ The synthesis is described in detail in the Supplementary Information (SI).

### Synthesis of polyethylene glycol-*block*-poly(ε-caprolactone)

The synthesis of the *block*-copolymer polyethylene glycol-*block*-poly(ε-caprolactone) (PEG-*b*-PCL) was performed following a previously reported procedure^31^ adapted from Meier et al*.*,^42^ which is described in more detail in the SI. The chemical identity of the synthesized PEG-*b*-PCL was confirmed by solution ^1^H-NMR spectroscopy (see SI, Figure S1).

### Synthesis of unstained and QD-encoded PSMPs via a polymerization procedure (route i)

The PSMPs were synthesized following a previously described polymerization procedure^31^, modified considering two other procedures reported by Kimura et al.^43^ and Nirmalananthan-Budau et al*.* (regarding COOH functionalization)^38^.

Typically, 400 mg of PVP were dissolved in 45 mL of a mixture of ethanol and water (9:1). Additionally, 45.75 mg of PEG-*b*-PCL were dissolved in 504 µL of toluene and placed on a shaker for 30 min. Both solutions were combined under argon atmosphere. The mixture was heated to 80 °C and stirred at 100 rpm for 30 min, before 5 mL of styrene (with or without QDs) and 180 mg AIBN dissolved in 5 mL of ethanol/water (9:1) were sequentially added. The reaction mixture was stirred at 80 °C for 3 h. Then, 150 µL of AA dissolved in 1.78 mL of ethanol/water mixture (9:1) were added dropwise to the reaction mixture. The reaction was continued for one hour before the mixture was cooled to room temperature (RT). The resulting particles were centrifuged at 1500 rcf for 2 min, the supernatant was discarded, and the particles were redispersed in 30 mL ethanol. They were again centrifuged (1500 rcf/2 min) and redispersed in 45 mL ethanol to create a stock solution. Prior to further investigation, the particles were washed three times with ethanol (also 1500 rcf/2 min) by discarding the supernatant after every step and redispersing the PSMPs in ethanol. The PSMPs were stored in ethanolic dispersion at RT.

For the QD incorporation during bead formation (polymerization), the CdSe/CdS-QDs were pretreated as follows: A spatula tip of OBDAC (approximately 2 mg) and 900 µL of ethanol were added to 100 µL of the QD dispersion in a vial. The mixture was homogenized on a shaker for 5 min, centrifuged at 6000 rcf for 5 min, and washed once with ethanol. The coated QDs were redispersed in 1 mL of styrene, sealed, and stored in the refrigerator. The polymerization procedure was performed as described above for the unstained PSMPs.

### Post-synthetic swelling procedure for QD loading (route ii)

2 mg of unstained PSMPs were dispersed in 1 mL of MilliQ water, washed twice with butanol, and finally redispersed in 500 µL of butanol. 5 µL of QDs (in hexane, 32.9 mg/mL (Cd content, determined by AAS)) were mixed with 95 µL of chloroform and added dropwise to the particle suspension under stirring. The reaction mixture was stirred for additional 30 min at RT, before it was centrifuged at 18,000 rcf for 15 min. The separated particles were washed twice with ethanol and once with MilliQ-water (centrifugation 18,000 rcf/15 min, supernatant discarded each time and particles redispersed in the respective solvent), followed by redispersion in 1 mL of MilliQ-water or ethanol.

### Post-synthetic swelling procedure for dye loading

Loading of the unstained PSMPs with RITC and NR were performed following a previously described swelling procedure^41^. In a typical experiment, 12 mg of the unstained PSMPs were dispersed in 4 mL of MilliQ-water. In parallel, 0.54 mg (1.0 µmol) of RITC or 0.32 mg (1.0 µmol) of NR were dissolved in 0.4 mL of a THF:DMF (50:50 w%) mixture. The dye solution was added to the particle dispersion and the mixture was vigorously shaken at RT for 1 h. During the incubation, the particles were ultrasonicated four times (2 min, ultrasonic bath). After 1 h, 0.6 mL of MilliQ-water were added before the particle dispersion was centrifuged at 10,000 rcf for 30 min. The separated particles were washed three times with MilliQ-water (centrifugation 10,000 rcf/30 min, discarding of supernatant, and redispersion of particles each time), followed by redispersion in MilliQ-water.

### Scanning electron microscopy (SEM)

For the SEM micrographs of the PSMPs, obtained with a Philips XL30 ESEM using an acceleration voltage of 20/25 kV, the samples were drop-casted onto aluminum holders from diluted PSMP dispersions and dried under ambient conditions. The mean particle sizes and the corresponding size distribution were determined using the software ImageJ (Version: 1.52e, https://imagej.nih.gov/ij/).

### Transmission electron microscopy (TEM)

TEM measurements of the QD dispersion were performed using a JEOL JEM-2100F-UHR equipped with a field emission gun and operated at 200 kV. The QD samples were prepared on carbon-coated copper grids (Quantifoil, 400 mesh) via drop casting and drying under ambient conditions. The mean particle size and the size distribution were determined with ImageJ as described for the SEM samples.

### High-angle annular dark-field scanning transmission electron microscopy (HAADF-STEM)

The HAADF-STEM measurements of both QD-loaded PSMPs were carried out with a ThermoFisher Scientific Talos F200S TEM at 200 kV. The samples were prepared by drop-casting diluted dispersions of the respective PSMPs in ethanol onto lacey, carbon-coated copper grids (PELCO by Ted Pella, Inc., 400 mesh).

#### Dynamic light scattering (DLS) and zeta potential measurements

DLS and zeta potential measurements of the unstained and QD-stained PSMPs dispersed in MilliQ-water were carried out with a Zetasizer Nano ZS from Malvern Panalytical Ltd. (back scattering angle 173°), equipped with a 633 nm laser, at T = 25 °C in disposable folded capillary cells (DTS1070, Malvern Panalytical). For each sample, three independent measurements including several sub-runs were performed. For the DLS measurements, the hydrodynamic diameter based on the z-average and number distribution was used. The zeta potential was calculated from the particle electrophoretic mobility using the Einstein–Smoluchowski theory, with a refractive index of 1.46 assumed for polystyrene for both studies.

#### Atomic absorption spectroscopy (AAS)

The Cd(II) concentration of the QD dispersions was determined with an AA140 instrument from Varian Inc. with an oxygen/acetylene flame atomizer after dissolving the QDs in *aqua regia* overnight. Six standard solutions with varying Cd(II) concentrations were employed to create a calibration curve.

#### Fluorescence spectroscopy

The emission spectra of the CdSe/CdS-QDs in toluene and the QD-loaded PSMPs in ethanol were recorded at 350 nm excitation with a calibrated FSP920 fluorescence spectrometer from Edinburgh Instruments Ltd. in (10 × 10) mm quartz glass cuvettes (Hellma GmbH) at RT.

#### Integrating sphere spectroscopy

The photoluminescence quantum yield (PLQY) values for the QD-loaded PSMPs in ethanol and the QD dispersion in toluene were determined with a stand-alone Quantaurus integrating sphere setup (Hamamatsu Photonics K.K, absolute determination). The measurements were conducted in (10 × 10) mm long-neck quartz glass cuvettes (Hamamatsu Photonics K. K) using an excitation wavelength of 350 nm at 25 °C. The respective solvent was used as a blank for the QD dispersion, while for both QD-loaded PSMPs, a dispersion of the unstained PSMPs with similar bead concentration was employed as a blank.

#### Fourier-transform infrared (FTIR) spectroscopy

FTIR spectroscopy was performed in the transmission mode with a Vertex 70 FTIR spectrometer from Bruker. The dried PSMPs were pestled together with 100 mg dry KBr (dried for four hours at 110 °C, then stored in a desiccator until use) in two different concentrations and pressed into tablets. The particle amounts used for this procedure were 3.0/4.6 mg (6.76 × 10^8^/1.04 × 10^9^ particles) for unstained, unfunctionalized PSMPs, 4.5/6.6 mg (4.12 × 10^8^/6.04 × 10^8^ particles) for QD-loaded PSMPs prepared via the post-synthetic swelling procedure, and 2.9/5.3 mg (3.51 × 10^8^/6.42 × 10^8^ particles) for QD-loaded PSMPs prepared via the polymerization procedure. A pure KBr tablet was employed to record a blank or background spectrum. After an atmospheric compensation (H_2_O, CO_2_) and background correction (concave rubber band correction with 10 iterations and 64 baseline points), the spectra were normalized (vector normalization) to the CH bands from 2655.1 to 3216.6 cm^−1^.

#### Conductometric titration

Conductivity measurements providing the maximum (total) number of (de)protonable COOH groups were carried out with a Modul 856 conductometer (Methrom). Prior to the measurements, the PSMPs were dialyzed against Milli-Q water to remove contaminations from the particle synthesis. For the measurement, samples containing either 20 mg of unstained PSMPs or 20 mg of QD-loaded PSMPs in 80 mL of Milli-Q water were employed. For the complete protonation/deprotonation of the FGs, the conductivity of the samples was adjusted to 100 μS/cm with either HCl (10 mM) or NaOH (10 mM) as a starting point. Titration with the base or the acid was performed in 20 μL steps at RT under argon atmosphere to exclude CO_2_.

#### TBO assay

The colorimetric TBO adsorption/desorption assay previously reported by us^40^ was performed with some modifications. 5 mg (1 × 10^11^ particles) of unstained and QD-loaded PSMPs were washed twice with MilliQ-water and redispersed in 0.8 mL of MilliQ-water. The pH of the particle suspensions was set to 8 and a solution of 0.2 mL of TBO (3.2 µmol) in MilliQ-water was added and incubated under gentle shaking for 20 min at RT. Subsequently, the particles were washed by several cycles of centrifugation, removal of supernatant, and addition of MilliQ-water. When the supernatant was clear, 1 mL of 1% SDS was added, and the particles were additionally incubated for 30 min under gentle shaking at RT. After separation of the supernatant from the particles, an absorption spectrum of the supernatant was measured and the absorbance at 632 nm (ε = 50,000 L mol^−1^ cm^−1^) was utilized to determine the amount of desorbed TBO, assuming one TBO molecule react with one COOH group. Finally, the particles were extensively washed to remove SDS, dried *in vacuo*, and the weight of the remaining particles was determined. This final step of particle drying and weighing accounts for partial loss of material during the excessive washing steps and reduces the variation coefficient to around 15%^40,44^.

#### *N*-APPA assay

*N*-APPA was freshly prepared as described in the literature^37,38^, and used for the catch-and-release assay. 5 mg (0.002 nmol/L) of unstained PSMPs, 5 mg (0.002 nmol/L) of QDs-loaded PSMPs (polymerization procedure), and 1 mg (0.0004 nmol/L) of QDs-loaded PSMPs (swelling procedure) were dispersed in 0.8 mL of MES buffer (pH 5.0), respectively. An excess of EDC (3 × 10^−11^ mol in 500 µL of MES buffer (pH 5.0)) and sulfo-NHS (3 × 10^−11^ mol in 500 µL of MES buffer (pH 5.0)) was added to each particle suspension and stirred at RT for 1 h. The activated particles were centrifuged and redispersed in 950 µL of phosphate buffer (pH 8.0), before 50 µL (0.2 µmol in MeOH) of *N*-APPA were added. The reaction mixture was shaken (600 rpm) overnight at RT, followed by centrifugation at 5000 rcf for 10 min, separation of the supernatant, and washing of the particles with 500 µL of phosphate buffer (pH 8) twice. The washed particles were redispersed in 450 µL of phosphate buffer and 50 µL (2 µmol) of a prepared TCEP solution in phosphate puffer were added, before the mixture was incubated for 45 min at RT. To separate the formed 2-thiopyridone (2-TP) from the particles, the mixture was centrifuged at 5000 rcf for 10 min and washed twice with 500 µL phosphate buffer (pH 8). The amount of 2-TP in the merged supernatants was photometrically quantified at 343 nm (ε = 8000 L mol^−1^ cm^−1^) as previously described^37^.

## Results and discussion

**Figure 1:**
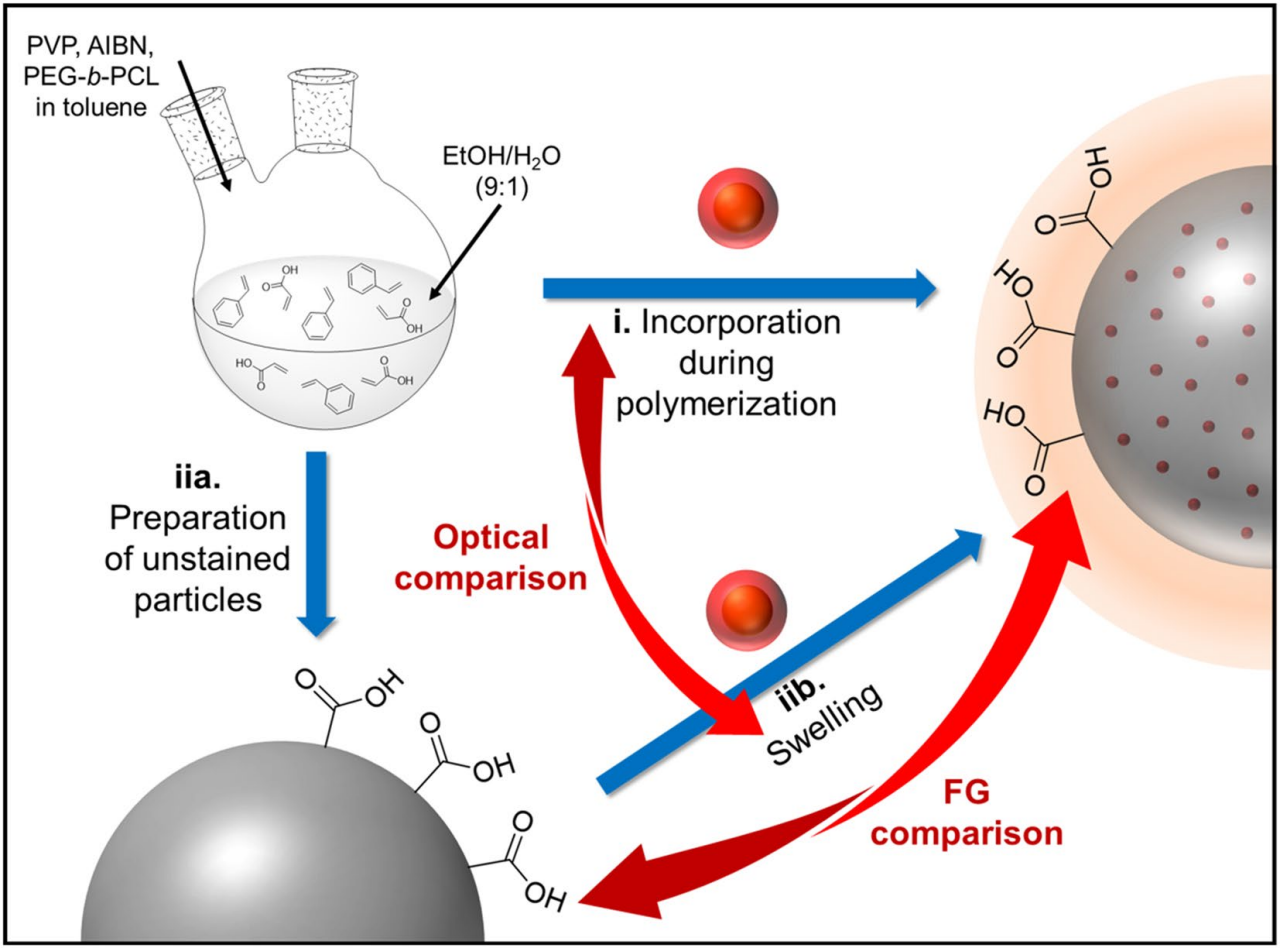
Schematic presentation of the synthesis routes used for the fabrication of carboxylated, QD-loaded polystyrene microparticles (PSMPs). (i) QD incorporation during the polymerization reaction (polymerization procedure) and (iia). preparation of unstained PSMPs followed by (iib) QD staining via a post-synthetic swelling procedure of the beads through addition of an organic solvent like butanol in presence of the QDs.

For the incorporation of the oleic acid and oleylamine stabilized CdSe/CdS-QDs into the COOH functionalized PSMPs, two different synthesis routes were utilized and compared. As schematically depicted in Fig. 1, this included i) QD addition during polymerization/bead formation (called polymerization procedure) and ii) a post-synthetic swelling procedure. For QD incorporation during bead formation, the QDs were dispersed in styrene and added during the polymerization procedure^31^. Thereby, the QDs are exposed to harsh reaction conditions, such as an elevated temperature and the presence of radicals, which can also affect their fluorescence properties. For the bead swelling procedure, first unstained, carboxylated microbeads were synthesized with a polymerization procedure previously established and adapted for the surface functionalization with COOH groups^31^. These unstained, pristine beads were also employed as control or blank to determine the influence of QD staining on the bead surface FGs. Bead encoding by a swelling and shrinking step was utilized by us before to encode differently sized and surface functionalized polymer beads with organic luminophores and sensor dyes^45–47^. This approach has been also pursued by other research groups to fabricate polymer particles, encoded with QDs^48–50^, other luminescent nanocrystals such as lanthanide-based nanomaterials^51,52^ or magnetic nanoparticles^53^ for use as carrier beads for bead-based assays, immune-separation or particle reporters for immunoassays. For QDs, particularly the organic solvent used for bead swelling must be carefully chosen as the solvent can induce quenching of QD luminescence.

Subsequently, we examined the influence of these two widely applied fabrication procedures of NP-encoded beads on the number or density of the surface FGs of the resulting QD-stained, COOH-functionalized PSMPs, as well as on the luminescence properties of the QDs incorporated into these PSMPs. We compared them to the respective pristine, carboxylated PSMPs and dye-stained, carboxylated PSMPs (utilizing RITC and the neutral polarity probe NR and the post-synthetic swelling procedure).

### Physico-chemical characterization of the (free) QDs

The CdSe/CdS-QDs were analyzed by TEM regarding their size (particle diameter: d_TEM_ = 10.3 ± 1.2 nm) and by AAS regarding their Cd(II) concentration (AAS: 32.9 mg/mL). Moreover, spectroscopic measurement were carried out (λ_em_ = 638 nm, PLQY = 58%).

### Physico-chemical characterization of the PSMPs

**Figure 2:**
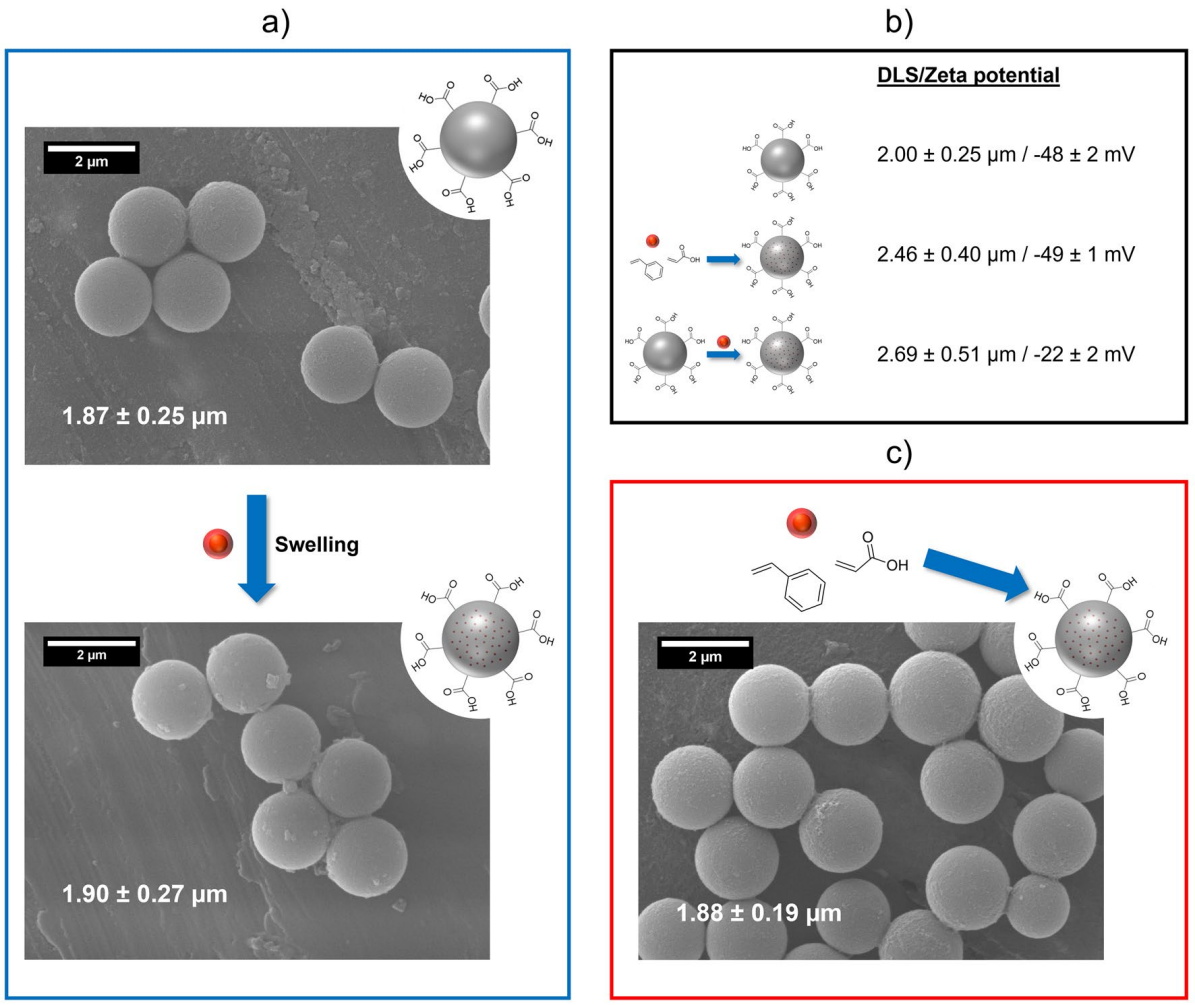
SEM images of the synthesized PSMPs, with (**a**) unstained PSMPs and QD-loaded PSMPs prepared from the former via the post-synthetic swelling procedure, and (**c**) QD-loaded PSMPs prepared via the polymerization procedure; (**b**) PSMP sizes determined by DLS and their zeta potential values, illustrated with schematic displays of the particle synthesis routes.

Subsequently, we compared and evaluated the properties of the QDs-loaded PSMPs prepared by the two different synthesis routes as well as the unstained PSMPs. For this reason, the size of all particles was first determined by DLS and SEM measurements. The corresponding results are displayed in Fig. 2, and the size distribution histograms are presented in the SI (see Figure S3).

The bead sizes obtained by DLS, corresponding to the intensity-based hydrodynamic diameter of the particles, differ between the three PSMP samples. This is most likely caused by the organic shell and hydration layer around the PSMPs changing through loading the particles with the QDs, and possibly also by a slightly changed refractive index also caused by the QD presence. The SEM micrographs show that all beads are nearly identical in size and have a spherical shape with slightly rough surfaces. Both particle size and morphology are barely influenced by the QD loading procedure. For the PSMPs prepared by the post-synthetic swelling procedure, some cases of particle fusion could be observed, as well as a rougher particle surface. These particles also showed a slightly larger size and size distribution than the other PSMPs, which can be attributed to the swelling process. The zeta potentials of the unstained and the QD-loaded polymer particles obtained by the polymerization procedure closely match with values of − 48 mV and − 49 mV. In contrast, the swelling procedure affects the zeta potential of the resulting QD-loaded PSMPs, which revealed a zeta potential of − 22 mV. A change in zeta potential is in good agreement with previous results obtained with dye-loaded polystyrene NPs and MPs using a similar swelling procedure, that also indicated an increase of the zeta potential after the dye-loading of around 10 mV (used dyes/particles: a dyad dye and a rhodamine-B derivate with self-manufactured polystyrene NPs and MPs, as well as NR with commercially available polystyrene NPs)^41,54^.

To determine the influence of the QDs on PSMP loading, similar studies were performed with the amphoteric dye RITC and the neutral polarity probe NR using our post-synthetic swelling procedure previously optimized for different classes of organic dyes (see also SI for more details)^46^. The resulting RITC- and NR-loaded PSMPs showed zeta potentials of − 39 mV and − 40 mV, respectively. These values are slightly higher than the zeta potential of − 48 mV of the unstained PSMPs. NR staining of commercial 2 µm-sized PSMPs, revealing a zeta potential of − 36 mV, resulted in dye-stained PSMPs with a zeta potential of − 33 mV. This shows that the large increase of the zeta potential in the case of the QD-loaded PSMPs cannot solely be explained by the swelling procedure. Apparently, also the presence of the QDs contributes to this effect. Reasons for this are discussed in later sections considering the location of the QDs in/on the PSMPs.

### Luminescence Properties of QD-loaded PSMPs

**Figure 3:**
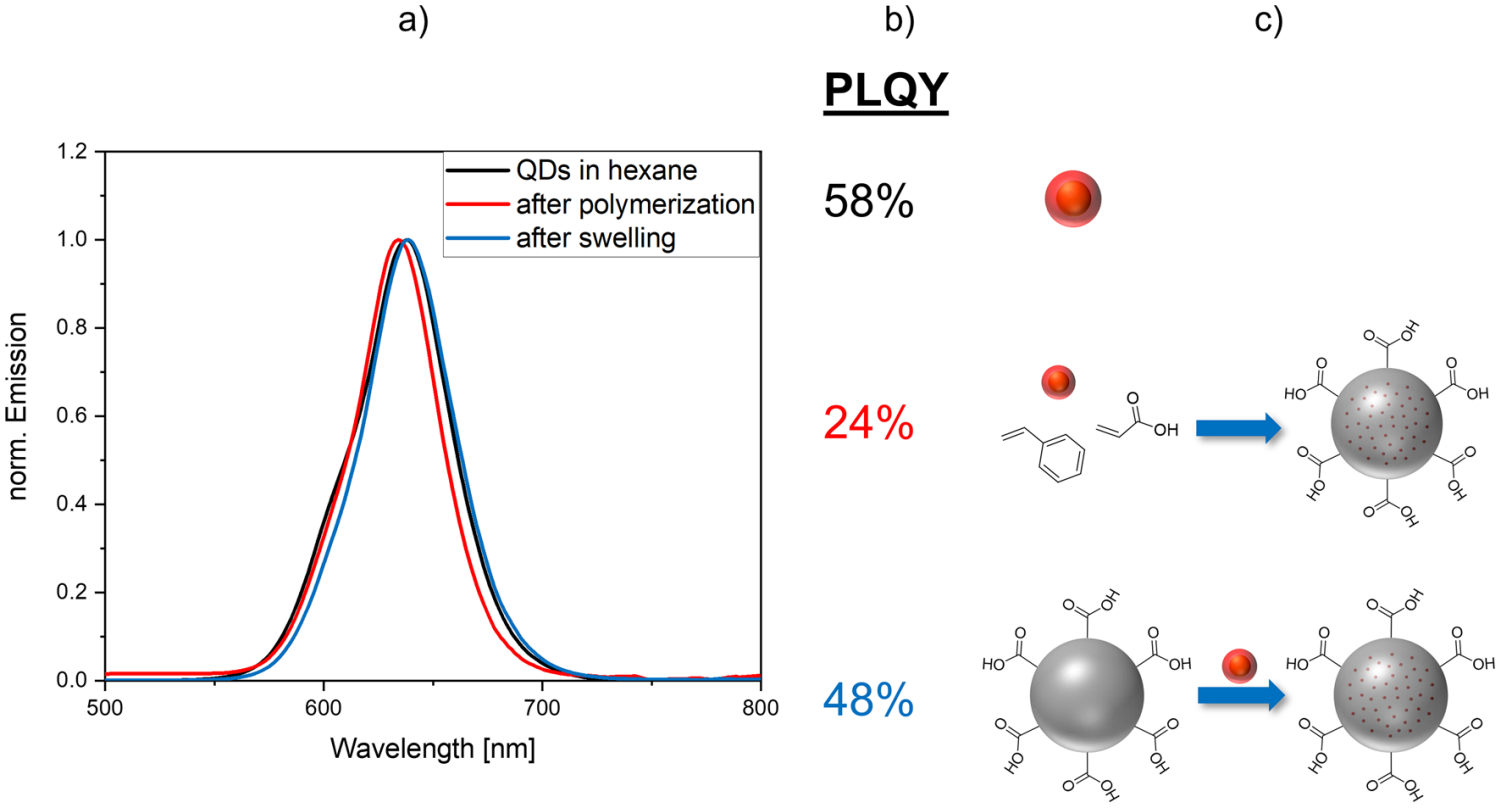
Optical characterization of the QD-loaded PSMPs. (**a**) Emission spectra of the CdSe/CdS-QDs dispersed in hexane and the QD-loaded PSMPs obtained by the polymerization and swelling procedures, respectively; (**b**) PLQY and (**c**) Schematic display of the synthesis routes leading to the respective PSMPs. The offset from zero of the red spectrum can be explained by scattering effects.

The luminescence properties of the QDs in the differently prepared PSMPs were compared by assessing their emission spectra and their photoluminescence quantum yields (PLQY), which is a measure for their photoluminescence efficiency. The former provides the spectral position and full width at half maximum (FWHM) of the QDs in the beads, which provide information on changes in QD size and size distribution. The latter indicates changes in QD surface chemistry, i.e., the formation of additional trap states during bead incorporation, favoring the non-radiative recombination of charge carriers. The corresponding spectra and PLQY values are displayed in Fig. 3. Apparently, the emission spectra of the QDs added during the polymerization procedure are hypsochromically shifted by about 4 nm compared to the emission spectra of the parent QDs dispersed in hexane. The swelling procedure only slightly affects the QD emission spectrum. Interestingly, the FWHM of the emission band of the QDs dispersed in hexane slightly exceeds the spectral bandwidths of the QD emission bands of the QD-loaded PSMPs. The refractive indices of hexane and polystyrene are slightly different (1.37 and 1.59, respectively), and their dielectric constants also differ (2.4-3 and 1.9). This can influence the QD emission features, as a change in QD environment can change, e.g., the emission maximum. The slight shift of the emission maximum that occurs only for the QDs added during the polymerization procedure, points to a slightly stronger interaction of the QDs with the polystyrene matrix as observed for the QDs incorporated with the swelling procedure. The narrowing of the FWHM in case of the QD-loaded PSMPs, compared to the free QDs, indicates a quenching of the smaller QDs during the preparation of these particles, leading to a narrowing in the energetically higher range of the emission spectrum. This can be explained by the reaction conditions to which the QDs are exposed in both loading procedures, which seem to affect and quench particularly the slightly smaller QDs.

For both staining procedures, the PLQY of the QDs decreases after PSMP incorporation. The decrease is more pronounced for the QDs present during the polymerization procedure. This diminution in PLQY is attributed to the elevated temperature and the presence of radicals during the polymerization reaction as well as to the presence of ethanol and water molecules. An ethanolic/aqueous environment is known to have a negative effect on the PL properties of some luminescent QDs by decreasing their stability e.g., by the removal of surface ligands or inducing irreversible aggregation and quenching^55^. However, the PLQY values of the QDs in both types of QD-loaded beads are sufficiently high for typical applications of such QD-encoded particles.

**Figure 4:**
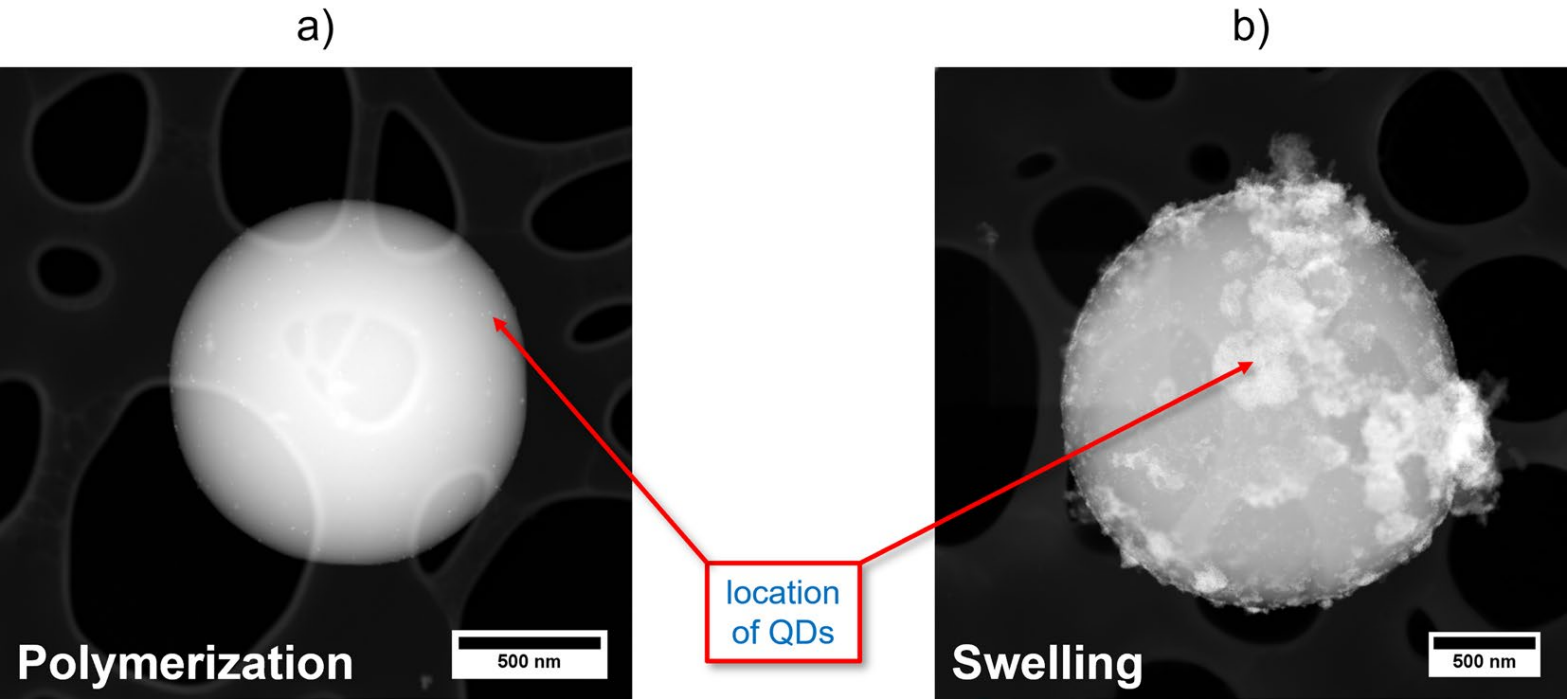
STEM images revealing the location of the CdSe/CdS-QDs (**a**) in QD-loaded PSMPs prepared by the polymerization procedure (route i) and (**b**) in QD-loaded PSMPs prepared by the post-synthetic swelling procedure (route ii). The differing amount of QDs visible (small, lighter particles on or close to the PSMP surface) is due to different QD concentrations used for the PSMP preparation.

Subsequently, the QD location and spatial distribution within the PSMPs prepared by the two different staining methods were explored by STEM measurements. The corresponding images are displayed in Fig. 4. These images reveal a QD accumulation at the PSMP surface, especially for the particles prepared by the post-synthetic bead swelling procedure. These findings are attributed to the direct binding of the ligand, i.e., oleic acid and oleylamine, stabilized QDs to the COOH groups on the PSMP surface. This seems to prevent QD penetration into the bead cores for the swelling procedure. In addition, we cannot exclude that the PSMPs do not swell enough to allow the QDs to properly and deeply penetrate the particles. The stabilization of the QDs with oleic acid also underlines the affinity of the QD surface atoms to carboxylic acid groups. We assume that during the reaction, some of the initial QD ligands detach from the QD surface and make room for the binding of the COOH groups located at the PSMP surface, resulting in the binding of the QDs to the PSMP surface FGs. In the case of the PSMPs prepared by the polymerization procedure, the QDs are located either on the particle surface or underneath the bead surface. In a previous study on QD encoding of PSMPs, utilizing very similar polymerization conditions with additional crosslinking of the polymer network with divinylbenzene, yet not a surface functionalization with carboxyl groups, we observed a QD accumulation in the PSMP core region (no significant agglomeration of the QDs)^31^. This indicates that the presence of acrylic acid with its COOH groups, and possibly also the absence of the crosslinker, prevent QD migration into the PSMP core. This finding can also explain why the zeta potential of the PSMPs is modified in the case of the swelling procedure, leading to an increase in zeta potential, contrary to the zeta potential of the PSMPs prepared by the polymerization procedure. In addition, it explains the observed spectral shift of the emission maximum of the PSMPs prepared by the polymerization procedure compared to the free QDs, which also underlines a stronger interaction of the QDs with the polystyrene matrix in this case. Such a spectral shift does not occur for QD-encoded PSMPs prepared by the swelling procedure. Apparently, for the homogeneous encoding of such polymer beads suitable for subsequent surface modifications, a two-step procedure could be better suited, preparing first plain QD-stained beads followed by the subsequent introduction of surface FGs.

**Figure 5:**
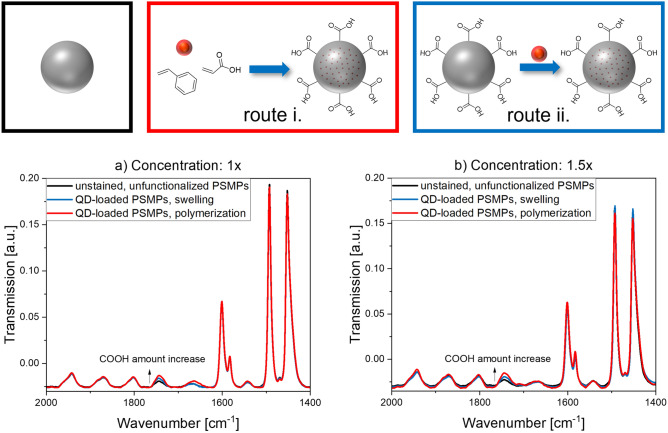
FTIR spectra (area of interest) of both QD-loaded PSMPs and unloaded, plain PSMPs, measured with two different PSMP concentrations, that reveal concentration and preparation-related changes in the intensity of the carbonyl peak at 1744 cm^−1^. The slight offset of the IR spectra (baseline value below 0) is caused by the normalization procedure. The complete IR spectra are provided in the SI (see Figure S4).

To study the potential influence of both QD staining approaches on the surface FGs of the resulting QD-encoded beads, the modified PSMPs were investigated with FTIR spectroscopy. This method should enable the semiquantitative determination of the amount of COOH FGs on the PSMPs. Therefore, the FTIR spectra of two different concentrations of both types of QD-loaded PSMPs and unstained, plain PSMPs (not functionalized with COOH FG, bead synthesis without acrylic acid), were measured. The IR spectra obtained for both concentrations are displayed in Fig. 5. From the spectra, we could determine whether the change in COOH amount can be detected by examining the vibrational bands caused by the carbonyl group. In this case, the OH band around 3350 cm^−1^ could not be used for the comparison, because it was not strong enough to be visible in the spectra (see also SI, Figure S4). This could be caused by a broadening of the OH band, which is common especially in the presence of (traces of) water, and additionally by the much stronger signals from the (poly)styrene overriding the OH signals. Resulting from the monomer ratio, the amount of Poly-AA (containing the COOH FGs) in the beads is much smaller than that of PS. Apparently, for the different PSMPs, there is a clearly visible and reproducible change in the intensity of the peak at 1744 cm^−1^, which is ascribed to carbonyl vibrations. For the plain beads, there is also a small peak present at this wavelength. This peak is attributed to the underlying, aromatic benzene vibrations, but the intensity of this peak is much less pronounced than those of the bands resulting for the functionalized PSMPs. All other bands in the IR spectra match after normalization. The higher intensities of the peak at 1744 cm^−1^ observed for both concentrations of the QD stained beads prepared by the two encoding methods indicate a higher amount of COOH surface groups for the PSMPs synthesized with the QDs present during bead formation.

Subsequently, the amount of surface FGs on the QD-loaded PSMPs and the similarly prepared unstained PSMPs was quantified by different analytical methods. For the quantification of the total number of COOH FGs, a conductometric acid/base titration was applied. As electrochemical titrations utilize the smallest possible reporters, namely protons (H^+^) and hydroxide ions (OH^−^), for signal generation, a maximum number of accessible FGs is detected with this approach. This number typically corresponds to the total number of (de)protonatable surface groups. This has been confirmed by us exemplarily for carboxylated polymer particles of different size by cross validation with ^13^C NMR measurements, i.e., by comparing the results of conductometric measurements and ^13^C NMR data^40,56,57^. As such, electrochemical titration methods are sensitive to the presence of (de)protonable and ionic contaminations present in the bead dispersion, remaining from particle synthesis like polymerization initiators, stabilizers, and salts, prior to conductometric measurements. Hence, all bead dispersions were purified by dialysis. The amount of COOH groups on the bead surface was determined to 127 ± 3 nmol/mg for the unstained PSMPs and to 155 ± 18 nmol/mg and 177 nmol/mg for the PSMPs, QD-stained via the polymerization and swelling procedure, respectively. This indicates an increase in the amount of COOH FGs for both staining procedures, particularly for the swelling procedure. Considering the STEM results, the higher total amount of COOH groups can be ascribed to the oleic acid ligand shell of the CdSe/CdS QDs that are located on the PSMP surface. This also explains why the total COOH amount on the surface of the PSMPs prepared via the swelling procedure is higher than the amount of COOH FGs found for the PSMPs prepared with addition of the QDs present during bead formation, as more QDs were used for the swelling procedure. This assumption is further supported by the results obtained for beads stained with the dye RITC shown in the SI (Figure S5). For RITC staining, a decrease of the amount of COOH FGs compared to unstained beads was obtained, as the dye does not introduce more COOH groups, yet can bind to existing COOH FGs on the polymer beads.

**Figure 6:**
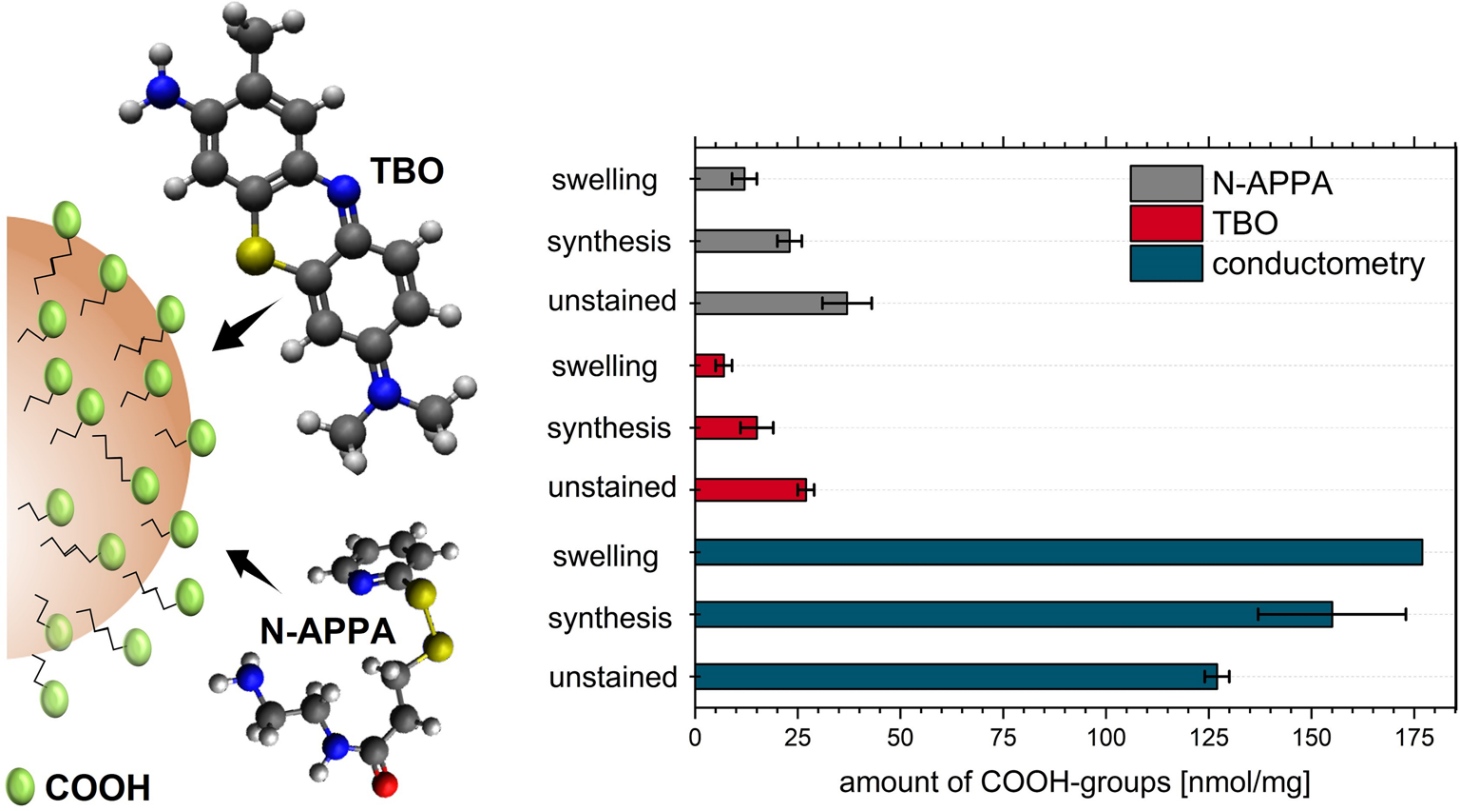
Quantification of the total and accessible amount of COOH groups on unstained and QD-loaded PSMPs (polymerization (here synthesis) and swelling procedure), using conductometry and optical assays with TBO and the cleavable reporter *N*-APPA. Both photometrically readout reporter molecules differ in size, shape, and steric demand with respect to the surface area and FGs to be detected.

Finally, the amount of accessible COOH groups on the unstained and the two types of QD-loaded PSMPs was determined (see Fig. 6 for results). Therefore, two optical assays were utilized that rely on the signal generation by optically detectable reporter molecules of different size and differ in the type of interaction/binding of the reporter to the particle surface. Both assays are versatile and allow for the quantification of the number of accessible surface groups on all types of transparent, scattering, absorbing and/or fluorescent particles as the actual optical quantification is performed in the supernatant after particle removal by centrifugation. Thereby, a distortion of the optical measurements by scattering is avoided. The colorimetric TBO assay exploits the adsorption/desorption of the positively charged dye TBO onto the surface of oppositely charged particles like negatively charged carboxylated beads. It yielded an amount of accessible COOH groups of 15 ± 4 nmol/mg COOH FGs and 7 ± 2 nmol/mg for the PSMPs stained with QDs by the polymerization and the swelling procedure, respectively. These numbers equal less than 20% of the total number of COOH groups found by conductometry. Please note here that the size of a TBO molecule with its three aromatic rings considerably exceeds the size of a COOH group. Hence, assuming a one-to-one binding stoichiometry of FG and dye considerably underestimates the accessible number of COOH surface groups. For a more reliable result, a stoichiometry factor must be used, which can be derived from a method comparison or cross validation. For example, in a previous study on the determination of the amount of COOH groups on polymethylmethacrylate (PMMA) beads grafted with different amounts of polyacrylic acid, we obtained a stoichiometry factor of 3.4 ± 0.2 by such a method cross validation^40^. An estimation of the theoretical steric demand of TBO on the particle surface (see Fig. 6, left, for molecule structures) indicates a maximum amount of about 6 nmol/mg PSMPs, depending on the assumption made on the binding/orientation of the TBO dye to the particle surface.

In addition, we performed a catch-and-release assay with the cleavable reporter *N-*APPA. *N-*APPA is first covalently bound to the COOH FGs to be quantified, followed by the reductive cleavage of the disulfide linker, yielding the photometrically detectable reporter 2-thiopyridone (2-TP). 2-TP is then photometrically quantified in the supernatant after particle removal by centrifugation. This versatile assay has been successfully used by us, e.g., for the quantification of different FGs on polymer and silica particles^37,38^. The size and hence the steric demand of *N*-APPA is smaller than that of TBO. Due to the less rigid molecular structure and smaller size, a higher number of accessible COOH groups should be found by this assay. As to be expected from these considerations, the data in Fig. 6 reveal 1.4–1.7 times higher values than obtained for the TBO assay. These results also underline the possible influence of the reporter size and shape on the quantification of the amount of accessible surface FGs and the importance of size and steric considerations for subsequent bead surface modifications with ligands or biomolecules^58^. The values of 23 ± 3 nmol/mg, 12 ± 3 nmol/mg, and 37 ± 6 nmol/mg as found for the QD-loaded PSMPs obtained by the polymerization and the swelling procedure and the unstained PSMPs, respectively, also exceed the calculated amount of *N*-APPA molecules that can cover the bead surface (10 nmol/mg). This confirms the trend observed for the TBO assay. The observation can possibly be ascribed to a surface penetration of the two optically read out dyes through the somewhat porous PSMP surface, that differs between both dyes. Thereby, not only COOH groups present on the particle surface, but also COOH FGs near the PSMP surface are detected. In addition, the maximum calculated amount of attached dye relies on the assumption of a very smooth PSMP surface, which is not completely true. This can also lead to the higher values for the synthesized PSMPs compared to the theoretical, accessible COOH amount.

For the different PSMPs, the trend for the accessible amount of COOH groups determined from both assays, yielding the highest amount of accessible COOH groups for the unstained particles, is reversed compared to the total amount of COOH groups obtained by conductometric titration. This can also be ascribed to the presence of the QDs on the PSMP surface. Here, two effects need to be considered. First, the oleic acid ligands on the QD surface bear COOH groups. These are coordinatively bound to the QD surface, but can be still protonated, and thus can be detected by the conductometric titration, but not by the considerably larger dye/reporter molecules. Hence, for the QD-stained PSMPs, these molecules do not contribute to the COOH amount measured with the optical assays. In addition, the COOH FGs on the QD-loaded PSMPs are partly occupied by the QDs and are thus not available for the interaction or covalent binding of the colorimetric reporters TBO and *N*-APPA. This can explain why the accessible COOH amount determined by both assays is highest for the unstained particles. Moreover, this can also explain why the accessible COOH amount found for the PSMPs prepared by polymerization in the presence of QDs exceeds the amount of accessible COOH FGs determined for the PSMPs prepared via the swelling procedure, as for the polymerization approach, fewer QDs were employed.

### Stability of the QD-loaded PSMPs

Subsequently, we performed first screening studies of the storage stability of both types of QD-loaded PSMPs and the unloaded PSMPs. The particles were all stored in ethanolic dispersion at RT in the dark. As application-relevant readout parameter/particle properties, the most relevant functional properties size, surface charge, and photoluminescence were used. After a storage period of four months, the particles were characterized utilizing DLS and zeta potential measurements, which are routinely employed for characterizing the colloidal stability of all types of NPs and MPs. In addition, fluorescence measurements were performed, which provide information on changes in the performance parameters particle size, surface charge, emission band position, and PLQY. The results of these measurements were then compared to the result of the initially performed PSMP characterization. The data are summarized in Table 1.

**Table 1:** Comparison of four months aged PSMPs with freshly prepared PSMPs, regarding particle size determined by DLS, zeta potential, emission spectra, and PLQY.

	Unstained PSMPs	QD-loaded, polymerization	QD-loaded, swelling
Particle size by DLS in µm			
After synthesis	2.00 ± 0.25	2.46 ± 0.40	2.69 ± 0.51
After four months	1.88 ± 0.15	1.78 ± 0.13	1.07 ± 0.17
Zeta potential by DLS in mV			
After synthesis	− 48 ± 2	− 49 ± 1	− 22 ± 2
After four months	− 40 ± 4	− 40 ± 1	− 24 ± 3
PLQY in %			
After synthesis	/	24%	48%
After four months	/	15%	31%
Emission spectra	/		

As follows from the DLS measurements summarized in Table 1, the particle size decreased for all PSMPs within four months. With a 60% decrease, the size difference is most notable for the QD-loaded PSMPs prepared via the swelling procedure. The QD-loaded PSMPs fabricated by the polymerization procedure show a decrease by about 18% and the size of the unstained PSMP size decreased only by about 6%. The zeta potential of the QD-loaded PSMPs prepared by the swelling procedure barely changed over time. In contrast, the zeta potentials of the unstained and QD-loaded PSMPs prepared by the polymerization procedure slightly increased from values of − 48/ − 49 mV to − 40 mV. This still indicates a good colloidal stability. Apparently, not only the presence of the QDs has a significant influence on PSMP stability, but also the synthesis route. The decrease in PSMP size suggests a partial disintegration of the particles over time, and the chosen synthesis route seems to have a significant influence on this process. Possible reasons, that are currently assessed by us, could be related to different amounts of QDs and/or surface groups. In this respect, also other factors will be examined in the future such as the solvent chosen for PSMP dispersion, i.e., ethanol, ethanol/water mixtures and water, as well as the storage temperature. Other factors, that could be relevant for particle stability, include the degree of purification, i.e., whether the particles were purified after synthesis or stored in the reaction mixture, the amount of surface FG, and the usage of crosslinkers for the polymer matrix. Interestingly, up to now, there are only relatively few data available on systematic long-term stability studies of PSMPs. For commercial polymer particles of comparable composition and made from different polymers, commonly water is used for particle dispersion and storage, but also ethanol and ethanol/water mixtures are used for this purpose. Particle manufacturers typically recommend storage in the refrigerator. The findings of Wilkinson et al.^59^, who performed stability studies with latex microparticles, reveal for example that the surfactant employed for particle synthesis can considerably affect particle stability. This is confirmed by first results from us regarding the deteriorating influence of the purification of the particles concerning polymer bead stability.

The luminescence properties of the QD-loaded PSMPs reveal a decrease in PLQY values from 24 to 15% and from 48 to 31% for the PSMPs prepared by the polymerization procedure and the swelling procedure, respectively. The emission spectra of both QD-loaded PSMPs underwent a hypsochromic shift. This shift is slightly more pronounced for the QD-loaded particles prepared via the swelling procedure. The decrease in PLQY and fluorescence intensity as well as the hypsochromic shift in fluorescence are attributed to time-dependent changes of the luminescence of surface-bound or near-surface located QDs, which were constantly exposed to ethanol used for PSMP dispersion and storage. Ethanol can initiate the quenching of the QD fluorescence, e.g., by removal of the QD surface ligands and/or irreversible QD aggregation, as shown by us in a previous publication^31^, resulting in a time-dependent loss in emission. To investigate the occurrence of QD leakage, which would also lead to a diminution in fluorescence intensity, the QD-loaded PSMPs stored for four months were centrifuged (2000 rcf/3 min). Subsequently, an emission spectrum of the supernatant was recorded (see SI, Figure S10). The spectrum revealed only very minimal, barely detectable leakage of the QDs. This confirms that QD leakage does not predominantly account for the observed loss in fluorescence intensity.

Please not that although both types of QD-loaded PSMPs show a significant decrease in QD fluorescence, they can still be used for many different life science applications after four months of storage under the here applied conditions. Moreover, the long-term stability of the PSMPs can most likely be considerably improved by optimized storage conditions, as described in the previous section.

## Conclusion and outlook

In summary, we prepared COOH surface-functionalized, quantum dot (QD)-loaded polymer microparticles (PSMPs) by two synthesis routes. These include QD addition during the polymerization procedure, and bead formation before QD loading via the postsynthetic swelling procedure. We utilized oleic acid and oleylamine stabilized CdSe/CdS-core/shell-QDs with a mean particle size of 10.3 ± 1.2 nm and a strong red luminescence as representative QDs. Subsequently, the application-relevant or functional properties of both types of about 2 µm-sized, QD-loaded PSMPs such particle size, amount of total and accessible surface functional groups (FGs), and photoluminescence were determined by different analytical and spectroscopic methods and compared. For this comparison, also unstained PSMPs as well as PSMPs stained with two differently charged molecular dyes were made and used as control samples. As revealed by this comparison, both synthetic approaches led to significant differences in the total and accessible amount of COOH groups on the surface of the PSMPs, as well as in the fluorescence properties of the PSMP-encoding CdSe/CdS QDs.

The main advantages and disadvantages of both synthesis routes are summarized in Table 2, also considering the suitability of the resulting carboxylated, QD-loaded PSMPs for future applications. Aside from the already mentioned differences in FG amount and luminescence properties, in terms of particle size, the chosen synthesis routes do not seem to have a significant effect on the PSMPs. With the polymerization procedure, however, the size can be more easily adjusted to specific needs, while for the swelling procedure, premanufactured PSMPs can be employed, which are commercially available. Another advantage of the former procedure is the higher yield of QD-encoded beads. Despite the differences in luminescence properties, FG amount, stability, and synthesis requirements/outcome, both QD-loaded PSMPs are suitable for a wide range of applications in the life sciences.

**Table 2:** Comparison of the two synthesis routes: QD-stained carboxylated PSMP prepared i.) with the QDs present during the polymerization procedure, and ii.) by a post-synthetic swelling procedure of carboxylated PSMPs.

Synthesis approach	Advantages	Disadvantages
(i) QD addition during the polymerization procedure (route i)	Large PSMP amounts (higher yield per synthesis batch)	More time consuming (in case of commercial particles)
	Higher amount of accessible FGs	No commercially available particles usable
	PSMP size easily adjustable	Less monodisperse
	Very versatile (size, crosslinking etc.)	Lower PLQY
	Better long-term stability	
(ii) QD-encoding by a post-synthetic swelling procedure of non-crosslinked, carboxylated particles (route ii)	Facile and good transferability to other systems (matrix & staining species)	Low PSMP amounts (lower yield per synthesis batch)
	Use of commercial particles	Lower amount of accessible FGs
	Potentially better monodispersity (use of commercial PSMPs)	Size restriction (premanufactured beads)
	Very good luminescence properties (esp. PLQY)	Reduced long-term stability
	Less time consuming (when using commercial particles)	

Overall, the results of our study highlight the possible influence of particle staining and loading, particularly on the number of accessible surface FGs, and hence on the subsequent conjugation of functional molecules. They provide a better understanding of the impact of the synthesis route on the application-relevant properties of the resulting luminophore-stained polymer particles. These insights can contribute to the reproducible preparation of safe(r) particles with an improved control of their surface functionalities. The surface functionalization largely determines the dispersibility, colloidal stability, and subsequent bioconjugation of these NP-loaded polymer particles, as well as their performance in specific applications^60,61^. These findings are also of considerable relevance for safe(r)-by-design concepts for nanomaterials that often involve the encapsulation of potentially hazardous and toxic nanomaterials in other as safer or safe regarded materials, such as certain polymers or micelles. In the future, we plan to expand these studies also to differently sized, i.e., smaller as well as larger nanoparticles and different surface chemistries, thereby also addressing parameters such as surface or particle charge.

In addition, the presented FG characterization can also be applied to other particle systems made from different materials. This includes, e.g., different types of QDs of varying morphology, not only II/VI semiconductors such as CdSe cores with different passivation shells, and iron oxide nanoparticles encapsulated in crosslinked micelles utilizing polymers such as functionalized poly(isoprene) and poly(isoprene)-*block*-poly(ethylene oxide), which bear COOH and amino FGs and are currently examined by us and other groups^12,62,63^. First studies of such systems utilizing the Fluram assay for the quantification of primary amino FGs suggest that this approach can discriminate between the amino groups of the organic micelle shell, which point outwards from the QDs and are available for (bio)functionalization, and the amino groups which are involved in QD coordination within the micelles. The latter are not accessible for subsequent derivatization.

## Supplementary Information

Supplementary Information.

## Data Availability

All data generated/analyzed during this study are included either in this article and its Supplementary Information files or are available upon request to the corresponding author (U. Resch-Genger, ute.resch@bam.de) or the first author (L. Scholtz, lena.scholtz@bam.de).
