# Supplementary material for: Influence of nanoparticle encapsulation and encoding on the surface chemistry of polymer carrier beads
Source: Sci Rep. 2023 Jul 24;13:11957. doi: 10.1038/s41598-023-38518-7 (PMC10366211; doi:10.1038/s41598-023-38518-7)
Supplement: Supplementary file 1 — Supplementary Information. [file 41598_2023_38518_MOESM1_ESM.pdf]

Supplementary Information for:

Influence of Nanoparticle Encapsulation and Encoding on the Surface Chemistry of Polymer Carrier Beads

Lena Scholtz[1,2], Isabella Tavernaro[1], J. Gerrit Eckert[3], Marc Lutowski[1], Daniel Geißler[1], Andreas Hertwig[4], Gundula Hidde[4], Nadja C. Bigall[3,5], Ute Resch-Genger[1,*]

[1]Federal Institute for Materials Research and Testing (BAM), Division 1.2 *Biophotonics*, Richard-Willstätter-Str. 11, 12489 Berlin, Germany.

[2]Free University Berlin, Institute for Chemistry and Biochemistry, Takustraße 3, 14195 Berlin, Germany.

[3]Leibniz University Hannover, Institute of Physical Chemistry and Electrochemistry, Callinstraße 3A, 30167 Hannover, Germany.

[4]Federal Institute for Materials Research and Testing (BAM), Division 6.1 *Surface Analysis and Interfacial Chemistry*, Unter den Eichen 87, 12205 Berlin, Germany.

[5]Cluster of Excellence PhoenixD (Photonics, Optics, and Engineering − Innovation Across Disciplines), 30167 Hannover, Germany.

*ute.resch@bam.de, Phone: +49 (0)30 8104 1134

1. Synthesis of CdSe/CdS-core/shell-QDs 2

2. Preparation and ^{1}H-NMR spectrum of polyethylene glycol-*block*-poly(ε-caprolactone) 2

4. Size distribution of PSMPs 4

5. FTIR spectra of PSMPs 4

6. Dye-loading of PSMPs by postsynthetic swelling procedure with rhodamine B isothiocyanate (RITC) and Nile Red (NR) 5

7. Conductometry 5

8. Investigation of QD leakage after four months of storage 7

1. Synthesis of CdSe/CdS-core/shell-QDs

The CdSe/CdS-QDs with a core/shell-architecture were prepared according to a previously described procedure[1] adapted from Carbone *et al.*, Nightingale *et al.* and Chen *et al.*. [2-4]

In the first step, CdSe cores with wurtzite structure were synthesised according to Carbone *et al.*[2] For this synthesis, 120 mg (0.93 mmol) CdO together with 560 mg (1.67 mmol) ODPA and 6 g (15.51 mmol) TOPO were degassed at 150 °C for 1 h. The mixture was then heated under argon flow to 300 °C. After the injection of 2 mL (4.48 mmol) of TOP, it was heated to 380 °C and, following a retention period of 10 min, 3.6 mL of a previously prepared TOP/Se solution (120 mg/3.6 mL) was swiftly injected. The temperature was allowed to rise to 380 °C again before the reaction was quenched by addition of 5 mL of ODE and cooled down to 70 °C in an air stream. During the cooldown period, 5 mL of toluene was added to prevent solidification. The resulting particles were precipitated by methanol/isopropanol (1:2), centrifuged at 6,000 rcf and redispersed in 2 mL of hexane.

A $Cd(oleate)_2$ precursor solution was synthesised according to Nightingale *et al.*[3] For this synthesis, a mixture of 1.284 g (1 mmol) CdO, 12.94 mL (40.77 mmol) of oleic acid and 7.04 mL of ODE was degassed for 10 min at 100 °C. The dispersion was heated to 180 °C under argon flow and kept there for 60 min under vigorous stirring. To remove water as a side product, the mixture was cooled to 120 °C and degassed for 45 min. The 0.5 M $Cd(oleate)_2$ solution was used as prepared for the next step.

The growth of the CdS surface passivation shell was performed according to an adapted synthesis by Chen *et al.*[4] For this, 100 nmol of the CdSe cores (60 – 100 µL) were dispersed in 3 mL of ODE and OLA, respectively. The mixture was carefully degassed for 30 min at 90 °C. In the meantime, the S and Cd precursor solutions were prepared. For the desired shell thickness of 10 monolayers, 3.191 mL of $Cd(oleate)_2$ and 286 µL of 1-octanethiol were diluted to a total volume of 7 mL with ODE, respectively. The flask was then heated under argon flow in two steps to 310 °C. When reaching 240 °C, the simultaneous injection of the previously prepared $Cd(oleate)_2$ and 1-octanethiol solutions via syringe pump (6 mL, 3 mL/h) was initiated. After two hours, 1 mL of oleic acid was injected, and the temperature was kept at 310 °C for another hour. Finally, the reaction mixture was cooled down to RT in an air flow, and the particles were precipitated by addition of acetone, centrifuged, and redispersed in hexane.

2. Preparation and ^{1}H-NMR spectrum of polyethylene glycol-*block*-poly(ε-caprolactone)

The *block*-copolymer polyethylene glycol-*block*-poly(ε-caprolactone) (PEG-*b*-PCL) was prepared according to a previously reported procedure[1] adapted from Meier *et al.*. [5]
For this, 800 mg of poly(ethylene glycol) was added to a dry flask together with 1536 µL(14.53 mmol) of ε-caprolactone. The mixture was placed in a preheated aluminium heating block and stirred for 5 min at 130 °C, followed by the addition of one drop of Sn(II) 2-ethylhexanoate as a catalyst and initiator. The mixture was then stirred at 130 °C for 3 h before it was rapidly cooled with an ice bath, leading to the precipitation of a white, solid product. The raw product was then recrystallized by first dissolving it in a small amount of dichloromethane, followed by precipitation with *n*-heptane. The such obtained *block*-copolymer was then filtered and washed several times with *n*-heptane before drying.
Characterization of the synthesized PEG-*b*-PCL was done by nuclear magnetic resonance spectroscopy (solution ^{1}H-NMR) at RT with a 400 MHz JEOL JNM-ECX400 spectrometer (Free University Berlin). The sample was prepared by dissolving 6 mg of PEG-b-PCL in 700 µL of $CDCl_3$.

Chemical shifts: ^{1}H-NMR ($CDCl_3$, 400 MHz): δ = 1.39 (m, 2H, γ), 1.63 (m, 4H, β & δ), 2.30 (m, 2H, α), 3.63 (s, 4H, a & b), 4.05 (t, 2H, ε), 4.22 (t, 2H, b).

The number-average molecular weight M_n of the synthesized PEG-*b*-PCL was determined to be about 8024 g/mol according to Meier *et al.* from the ratio of protons corresponding to the PEG and PCL signals.[5]

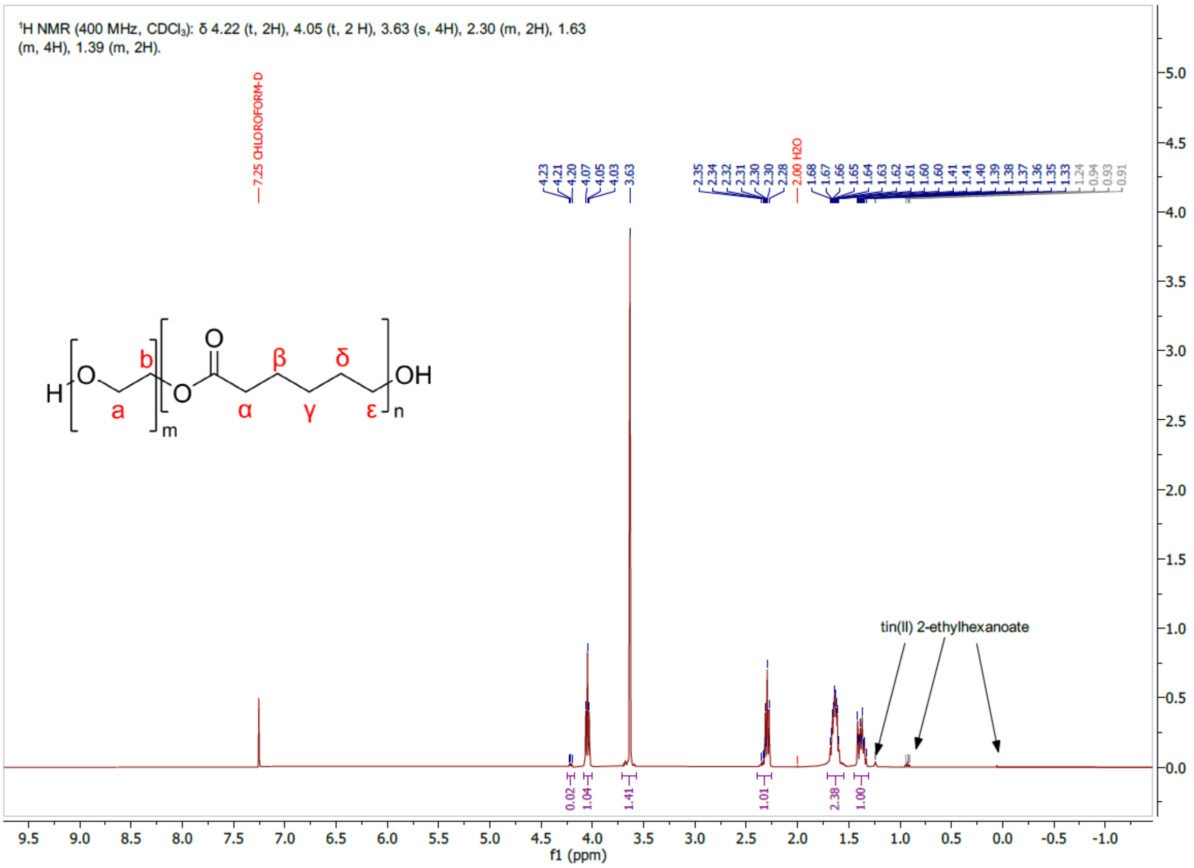

Figure S1: ^{1}H NMR spectrum (400 MHz, $CDCl_3$) with structural formula of the synthesized PEG-*b*-PCL, including all compound peaks, solvent peaks and three impurity peaks that can be attributed to the catalyst Sn(II) 2-ethylhexanoate.

3. Electron microscopy of CdSe/CdS-QDs

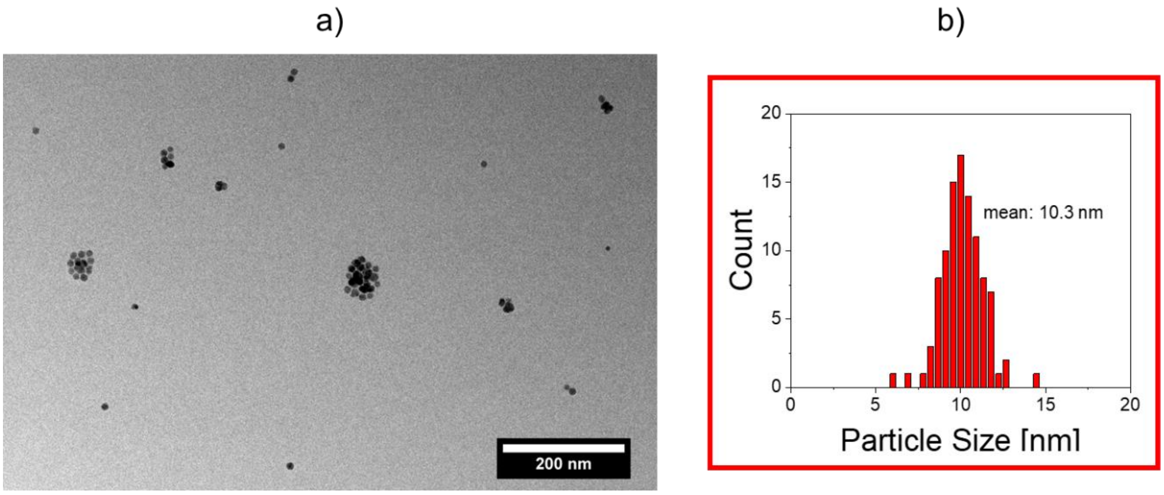

Figure S2: a) TEM image and b) corresponding histogram of the particle size distribution of the CdSe/CdS semiconductor core/shell-quantum dots (QDs).

The mean particle size of the QDs was calculated from TEM images to be 10.3 ± 1.2 nm. The PLQY was determined to be 58% in hexane, and the Cd concentration of the QD dispersion was determined by atomic absorption spectroscopy (AAS) to be 32.85 mg/mL.

4. Size distribution of PSMPs

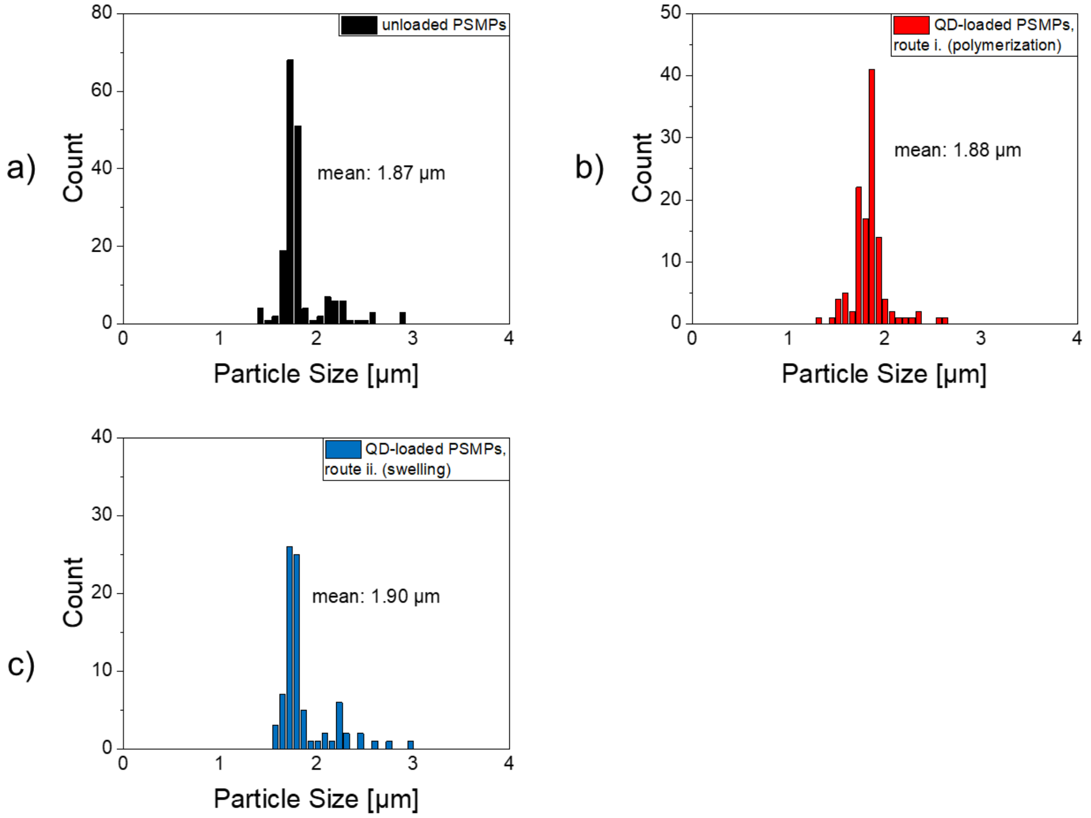

Figure S3: Histograms of the particle size distributions resulting from TEM micrographs of a) unloaded PSMPs, b) QD-loaded PSMPs prepared with QDs present during polymerization (route i.), and c) QD-loaded PSMPs prepared with a post-synthetic swelling procedure (route ii.).

5. FTIR spectra of PSMPs

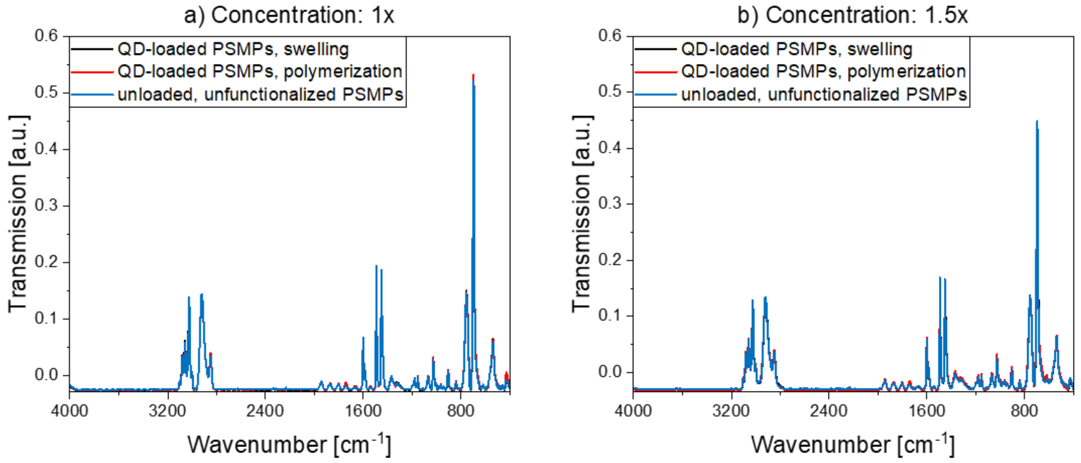

Figure S4: Full range FTIR spectra of both QD-loaded PSMPs and unloaded, unfunctionalized PSMPs with a change in carbonyl peak intensity, measured with two different PSMP concentrations. The slight offset of the spectra (baseline value below 0) is caused by the normalization procedure, the peak deviation at the right end of the spectra can be ascribed to impurities.

6. Dye-loading of PSMPs by postsynthetic swelling procedure with rhodamine B isothiocyanate (RITC) and Nile Red (NR)

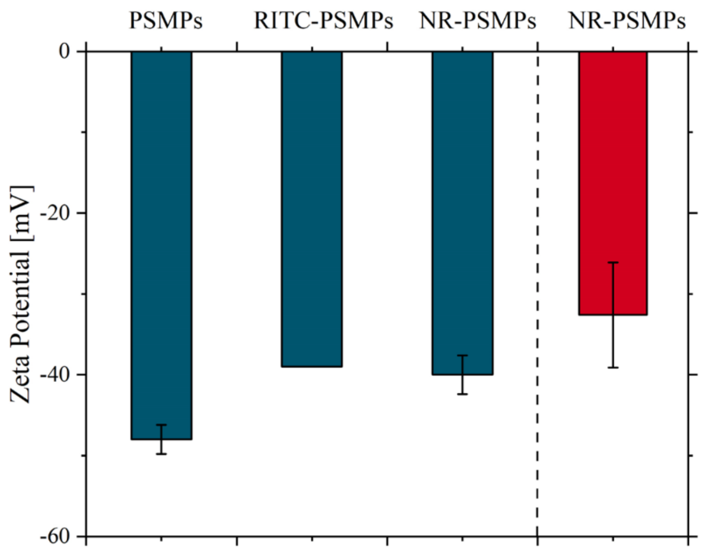

Figure S5: Comparison of zeta potentials of synthesized (blue) and commercially available 2 µm PSMPs (red), loaded with RITC and NR in a postsynthetic swelling step.

7. Conductometry

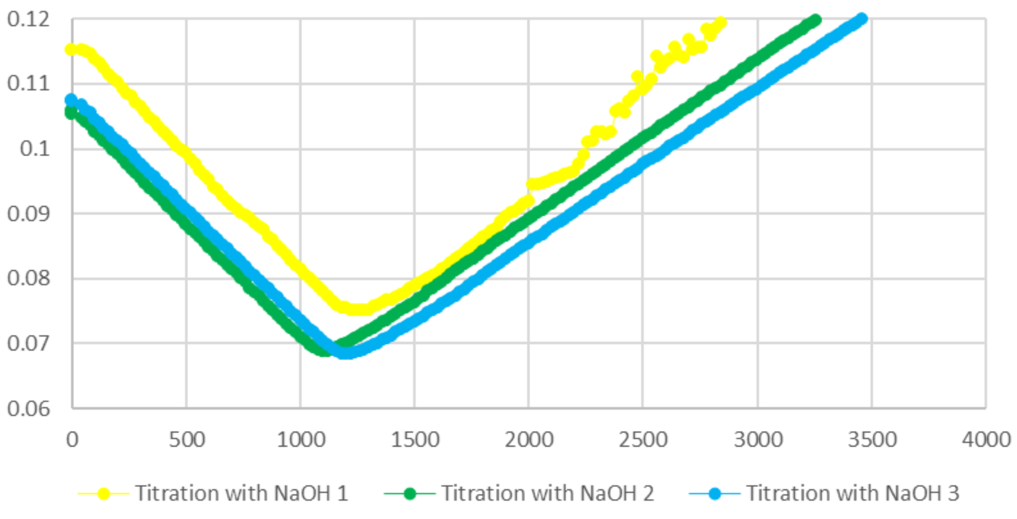

Figure S6: Results of conductivity measurements of unstained PSMPs.

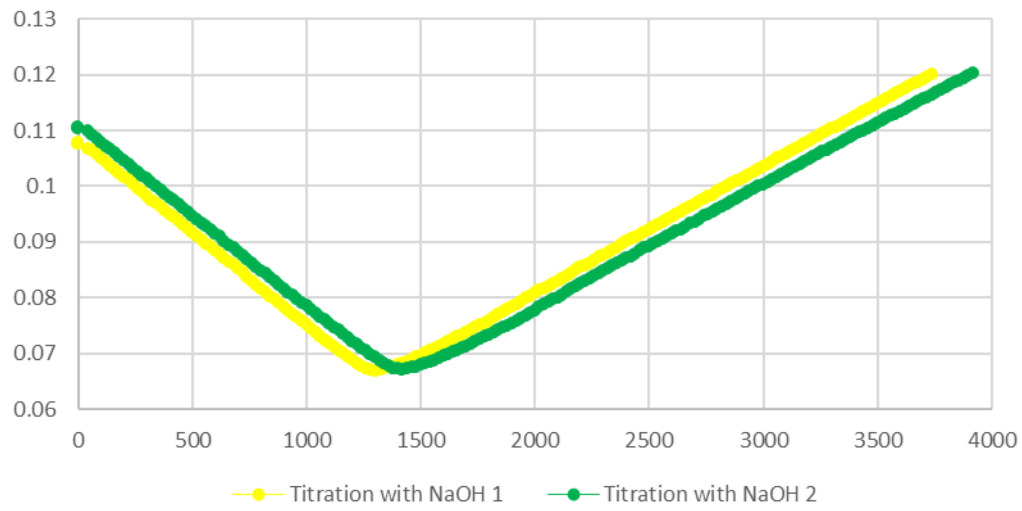

Figure S7: Results of conductivity measurements of QD-loaded PSMPs, prepared by the polymerization procedure.

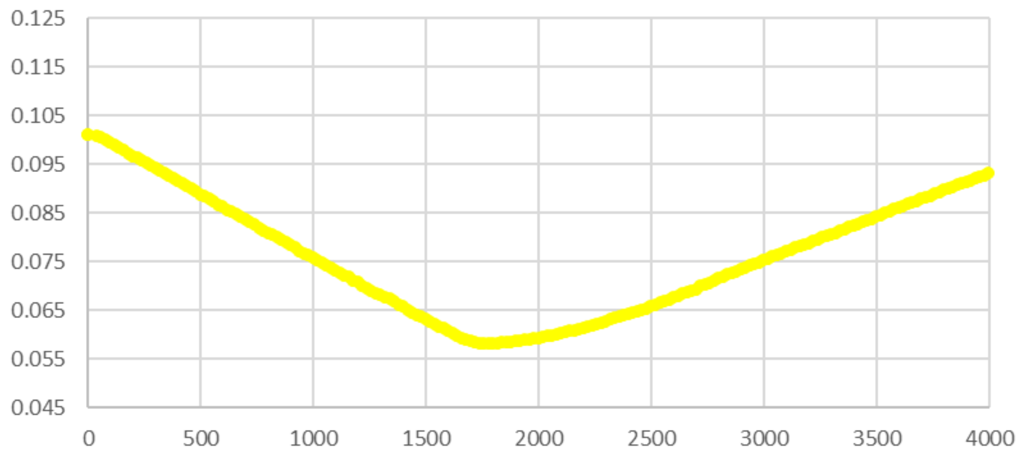

Figure S8: Results of conductivity measurements of QD-loaded PSMPs, synthesized by the postsynthetic swelling procedure.

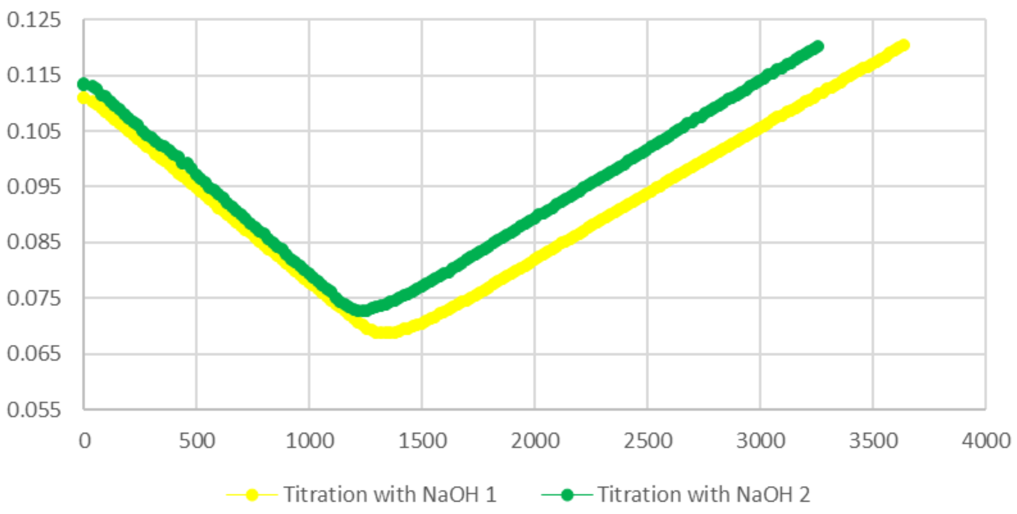

Figure S9: Results of conductivity measurements of RITC-loaded PSMPs, synthesized by the postsynthetic swelling procedure.

8. Investigation of QD leakage after four months of storage

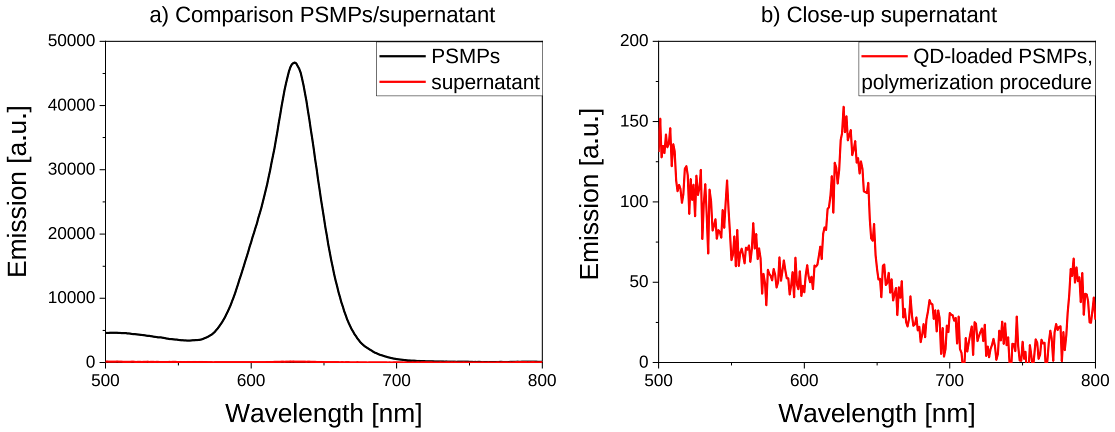

Figure S10: a) Emission spectra of QD-loaded PSMPs (prepared by the polymerization procedure) and their supernatant after centrifugation (2,000 rcf/3 min), with b) a close-up on the supernatant emission.

References

(1) Scholtz, L.; Eckert, J. G.; Elahi, T.; Lübkemann, F.; Hübner, O.; Bigall, N. C.; Resch-Genger, U. Luminescence encoding of polymer microbeads with organic dyes and semiconductor quantum dots during polymerization. *Scientific Reports* **2022,** *12* (1), 12061.

(2) Carbone, L.; Nobile, C.; De Giorgi, M.; Sala, F. D.; Morello, G.; Pompa, P.; Hytch, M.; Snoeck, E.; Fiore, A.; Franchini, I. R.et al. Synthesis and Micrometer-Scale Assembly of Colloidal CdSe/CdS Nanorods Prepared by a Seeded Growth Approach. *Nano Letters* **2007,** *7* (10), 2942.

(3) Nightingale, A. M.; Bannock, J. H.; Krishnadasan, S. H.; O'Mahony, F. T. F.; Haque, S. A.; Sloan, J.; Drury, C.; McIntyre, R.; deMello, J. C. Large-scale synthesis of nanocrystals in a multichannel droplet reactor. *Journal of Materials Chemistry A* **2013,** *1* (12), 4067.

(4) Chen, O.; Zhao, J.; Chauhan, V. P.; Cui, J.; Wong, C.; Harris, D. K.; Wei, H.; Han, H.-S.; Fukumura, D.; Jain, R. K.et al. Compact high-quality CdSe–CdS core–shell nanocrystals with narrow emission linewidths and suppressed blinking. *Nature Materials* **2013,** *12* (5), 445.

(5) Meier, M. A. R.; Aerts, S. N. H.; Staal, B. B. P.; Rasa, M.; Schubert, U. S. PEO-b-PCL Block Copolymers: Synthesis, Detailed Characterization, and Selected Micellar Drug Encapsulation Behavior. *Macromolecular Rapid Communications* **2005,** *26* (24), 1918.
